# IDENTIFYING SOLUTIONS TO ADDRESS THE BEHAVIORAL HEALTH OF OLDER ADULTS: A QUALITATIVE ANALYSIS

**DOI:** 10.1093/geroni/igad104.3408

**Published:** 2023-12-21

**Authors:** Allyson Stodola, Sheryl Elliott, Nathan Parsons, Sarah Dys, Susan Sotka, Walter Dawson

**Affiliations:** Portland State University, Portland, Oregon, United States; Portland State University, Portland, Oregon, United States; Portland State University, Portland, Oregon, United States; Portland State University, Portland, Oregon, United States; Institute on Aging, Portland, Oregon, United States; Oregon Health & Science University, Portland, Oregon, United States

## Abstract

The Oregon Older Adult Behavioral Health Initiative (OABHI), a program of the Oregon Health Authority, seeks to improve behavioral health (BH) services for older adults and people living with physical disabilities. Twenty-four OABHI Specialists work with BH and Aging services agencies, community-based organizations, and other key partners to strengthen the BH workforce, educate communities, provide complex care consultations, improve intra-agency collaborations, and foster timely access to coordinated, culturally-responsive services. Semi-structured, in-depth interviews with Aging and BH service agency managers and direct service providers (n=29) across Oregon were conducted over 12 weeks in 2023 to evaluate impacts of their involvement with the OABHI and improvements needed to better address the BH needs of these populations. Direct service staff and managers shared suggestions on programs, policies and inter-agency agreements that should be implemented to address the top BH-related challenges in their area. The first-hand experiences of direct service staff and managers who work with these populations provide unique insight into not only the most prevalent systemic issues, but what they perceive to be the key resources needed to help address these issues. Qualitative data were analyzed to develop systems-level recommendations to address gaps in BH resources for older adults, and were presented to policymakers and other decision makers in Oregon. Results indicate key resources and policy changes needed to support OABHI-delivered interventions, promote intra-agency collaboration, increase investment in BH services, and provide resources for an increasing population of older adults and people living with physical disabilities who have BH needs.

